# Differential metabolic responses to hydrogen peroxide-induced oxidative stress in parotid and submandibular gland acinar cell lines

**DOI:** 10.1016/j.bbrep.2026.102596

**Published:** 2026-04-17

**Authors:** Golnaz Golnarnik, Anni I. Nieminen, Tine M. Søland, Hilde K. Galtung, Trude M. Haug

**Affiliations:** a Institute of Oral Biology, Faculty of Dentistry, University of Oslo, Oslo, Norway; b Stem Cell and Metabolism Research Program, Faculty of Medicine, University of Helsinki, Finland

**Keywords:** Salivary glands, Parotid gland, Submandibular gland, Oxidative stress, Metabolomics, Succinic acid, Metabolites

## Abstract

This study explored how oxidative stress induced by hydrogen peroxide alters metabolite levels in submandibular and parotid salivary gland acinar cells. We investigated the impact of oxidative stress on the metabolite profiles of two salivary gland cell lines and examined differences in response. Rat-derived immortalized acinar epithelial cell lines from the parotid and submandibular salivary glands were exposed to 50 μM and 150 μM hydrogen peroxide for 24 h. Metabolite levels were then analyzed using liquid chromatography mass spectrometry, and succinate levels were independently assessed using a succinic acid quantification kit. We observed a metabolic divergence between the two cell types. Succinate, fumarate, malate, and aspartate levels were increased in parotid gland cells but decreased in submandibular gland cells. Conversely, NADPH levels increased in submandibular gland cells but remained below the limit of detection in parotid gland cells. Both cell types exhibited glutathione depletion and oxidized glutathione accumulation. Notably, submandibular gland cells exhibited a distinct metabolic shift in response to 150 μM hydrogen peroxide exposure. Submandibular gland cells demonstrated superior redox adaptability compared to the metabolic vulnerability observed in parotid gland cells. These findings provide a metabolic basis for the differential radiosensitivity of salivary glands and may inform biomarker discovery for monitoring and preserving gland function in oxidative stress-related diseases.

## Highlights

• Oxidative stress changed metabolite levels in two salivary acinar cell lines.
• Submandibular cells exhibited greater redox adaptability than parotid cells.
• Submandibular cells showed a distinct metabolic shift.
• Succinic acid was elevated in parotid but suppressed in submandibular cells.

## Graphical abstract

**Figure:**
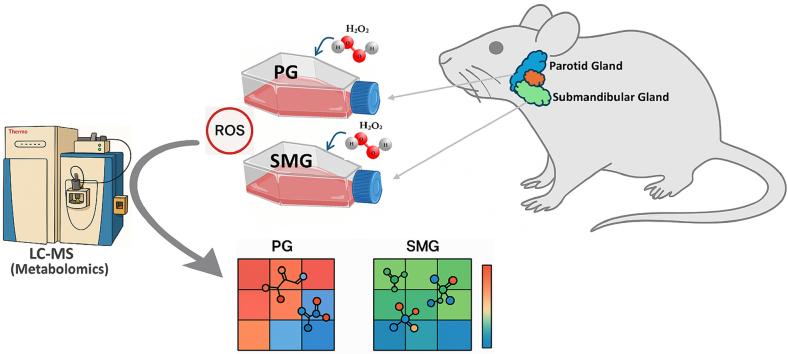

## 1 Introduction

Hyposalivation is reduced saliva production as a result of impaired function of the major salivary glands, including the parotid (PG), submandibular (SMG), and sublingual (SL) glands, as well as the minor salivary glands located throughout the oral cavity [1,2]. Insufficient saliva production can cause various complications, such as oral discomfort, difficulty in speaking and swallowing, altered taste perception, and increased risk of oral infections, all of which can significantly diminish an individual's quality of life [3]. These disorders are common in head and neck cancer patients receiving radiation therapy, as well as in patients diagnosed with, e.g., Sjögren's disease and diabetes. In addition, age-related changes in salivary gland function are related to hyposalivation. Oxidative stress plays a key role in the development of all these disorders, causing cell damage and contributing to salivary gland dysfunction and thereby hyposalivation [[4], [5], [6], [7]].

Reactive oxygen species (ROS), such as hydrogen peroxide (H_2_O_2_), are natural byproducts of aerobic metabolism primarily generated in the mitochondria [8,9]. These levels can be exacerbated by external stressors like radiation exposure and inflammation [10,11]. Such increases are observed in diseases like autoimmune disorders, diabetes, neurodegenerative conditions, and infections [[12], [13], [14]]. While physiological levels of ROS function as signaling molecules, their accumulation beyond the cell's antioxidant capacity results in oxidative stress [15,16]. This imbalance leads to irreversible damage to lipids, proteins, and DNA, and is a well-established driver of salivary gland dysfunction in conditions such as Sjögren's disease and post-irradiation injury [5,17].

Salivary glands are highly metabolically active tissues, requiring substantial ATP production to support continuous fluid secretion and protein synthesis [18]. This energy demand is primarily met through the tricarboxylic acid cycle (TCA), a key metabolic pathway that oxidizes nutrients to fuel the electron transport chain (ETC) and oxidative phosphorylation (OXPHOS) for ATP generation [19]. However, this reliance on aerobic metabolism makes the glands particularly vulnerable for oxidative stress and mitochondrial dysfunction [20,21]. Under stress conditions, the accumulation of TCA intermediates, specifically succinate, can switch from generating energy to driving pathologic ROS production, primarily via reverse electron transport (RET) at mitochondrial Complex I. Metabolites serve as important biomarkers of physiological and pathological conditions and may serve as early and differential diagnostic markers [22]. Potentially acting as such, they offer a critical tool for monitoring salivary gland dysfunction in irradiated patients and those with Sjögren's disease [[23], [24], [25]].

Although the PG and SMG share functional similarities, clinical and experimental data indicate a divergence in their susceptibility to stress [26]. The PG is consistently reported to be more radiosensitive and prone to irreversible dysfunction compared to the relatively resistant SMG. While the differential impact of stress on salivary glands is recognized, the specific metabolic adaptations that underpin their varying survival rates remain incompletely understood. Previous studies have shown that mitochondria in PG are significantly more susceptible to dysfunction and ROS generation under metabolic stress compared to those in SMG in high-fat diet mice [27]. However, a direct comparison of their dynamic metabolic flux and specific adaptive mechanisms is still required. Understanding these distinct metabolic profiles is crucial for uncovering why SMG cells survive oxidative insults that compromise PG function.

In a previous study, we showed that H_2_O_2_ triggers the production of intracellular ROS in acinar cell lines originating from rat SMG and PG, causing dysregulation of components in the salivary regulatory pathway [28] and changes in the cellular protein profile [29]. A similar dysregulation has been connected to hyposalivation in several studies [7,30]. To date, however, no study has explicitly compared the metabolic responses of PG versus SMG cells under oxidative stress. Therefore, the aim of this study was to identify and compare these distinct metabolic profiles using a liquid chromatography–mass spectrometry (LC‒MS) metabolomics approach. We aimed to elucidate the key metabolic pathways affected by oxidative stress and to identify potential biomarkers that could serve as early indicators of salivary gland dysfunction.

## 2 Materials and methods

### 2.1 Cell culture

Immortalized acinar epithelial cell lines, derived from the parotid (PG C10) and submandibular glands (SMG C10) of sexually mature male Sprague Dawley rats, were used for the experiments [31,32]. These cell lines are not commercially available and were generously provided by Professor Mary Reyland at the University of Colorado, USA. They have been authenticated and tested for mycoplasma within the project period (less than three years). Although these cell lines are of rat origin, they serve as robust in vitro models for human salivary gland dysfunction due to their stable phenotype and retention of functional acinar characteristics, including secretory granule formation and amylase activity, which are frequently lost in primary human cultures due to rapid dedifferentiation. The cells were cultured in Dulbecco's Modified Eagle Medium/Nutrient Mixture F-12 (DMEM/F12 50:50) (Gibco, Thermo Fisher Scientific, Massachusetts, USA). The growth medium was supplemented with 5 mM l-glutamine, 4 mg/mL insulin, 2.5% fetal bovine serum (FBS), 0.8 mg/mL epidermal growth factor (EGF), 0.1 μM retinoic acid, 10 mg/mL hydrocortisone, 5 mg/mL T3 (3,3′,5-triiodo-l-thyronine sodium salt), 1‰ trace element mixture (100x) (BioSource International, Camarillo, California, USA), and 50 μg/mL gentamicin. A humidified incubator with a 5% CO_2_ atmosphere was used to maintain all cultures at 37 °C. All reagents were from Sigma-Aldrich (St. Louis, MO, USA) unless otherwise noted. Experiments were conducted using cells from subculture numbers 10 to 15. Unlike the human SMG, a mixed seromucous gland, the rodent SMG is mostly serous, giving it a histological similarity to the PG [33]. Comparable growth rates and microscopic appearances were observed in both SMG and PG cultures.

### 2.2 Exposure to H_2_O_2_

A 50 mM stock solution of H_2_O_2_ was diluted in 5 ml of normal growth medium to produce final concentrations of 50 μM and 150 μM. Meanwhile, PG and SMG cells were seeded in T25 flasks at a density of 1.5 × 10^5^ cells/well using normal growth medium and incubated overnight. Notably, these concentrations and the 24 h exposure duration were selected based on our two previous studies, in which the same conditions were used to ensure experimental consistency and comparability of the results. Confluency reached 83% and 86% for PG and SMG, respectively. Fresh growth medium was added the following day; the experimental group received medium with two concentrations of H_2_O_2_, while the control group's medium contained no H_2_O_2_. The 24 h incubation period concluded with the removal and replacement of the cell's medium. After rinsing the cells with 5 ml of PBS, they were incubated in cold PBS for 1 min. Following scraping from the T25 flask, the cells were washed with 500 μl of PBS and collected in Eppendorf tubes. To pellet the cells, samples were centrifuged at 13,000×*g* for 5 min at 4 °C (using a Heraeus Fresco 17 from Thermo Fisher Scientific). The supernatant was then removed, and the remaining cell pellets, collected from three distinct seeding experiments, were stored at −80 °C to be used in the metabolomic analysis. The samples included biological replicates from different cell passages, each with a pair of technical replicates.

### 2.3 Targeted LC‒MS metabolic profiling

For Metabolite Extraction and LC-MS analysis the metabolites were extracted using a cold solvent mixture (Acetonitrile:Methanol:MQ; 40:40:20) followed by cycles of sonication, vortexing, and centrifugation [34]. Supernatants were evaporated under nitrogen and reconstituted in extraction solvent. Analysis was performed on a Thermo Vanquish LC system coupled to a Q-Exactive Orbitrap mass spectrometer (Thermo Fisher Scientific). Chromatographic separation was achieved on a SeQuant ZIC-pHILIC column (Merck) using a gradient of acetonitrile and ammonium hydrogen carbonate (pH 9.4). Data were acquired in full scan mode (55–825 *m*/*z*) with polarity switching, controlled by Xcalibur software. Metabolites were annotated using TraceFinder 5.1 against an in-house standard library. System stability was monitored using pooled quality control (QC) samples injected every 10th sample. Detailed chromatographic gradients, ionization voltages, and source parameters are provided in supplementary material (Tables S1 and S2).

For data processing post-acquisition, the data were filtered (RSD <20% in QC), normalized to total intensity, and missing values were imputed using the limit of detection (LoD) (1/5 of the minimum positive value for each variable). after which the data was log-transformed and autoscaled by mean-centering and dividing by the standard deviation of each variable.

### 2.4 Intracellular succinate measurement

Intracellular succinate levels were measured using a Succinate Assay Kit (#ab204718, Abcam, Cambridge, USA) according to the manufacturer's protocol. Briefly, 2 × 10^6^ cells/each sample (control, 50 μM H_2_O_2_, and 150 μM H_2_O_2_) were harvested and washed with cold PBS. Cells were subsequently resuspended in succinate assay buffer and homogenized quickly. Any insoluble material was removed by centrifugation. The collected supernatant was transferred to a new tube and subjected to succinate measurement. After 30 min of incubation in the reaction mixture, the absorbance was measured at OD 450 nm using an Epoch microplate spectrophotometer reader (Agilent BioTek, Santa Clara, California, United States).

### 2.5 Statistical analysis

Using GraphPad Prism for Windows (version 9.5.1, https://www.graphpad.com/), statistical analyses were conducted. All data are reported as the mean ± standard deviation (SD). Before group comparisons, the D'Agostino & Pearson test was applied to verify data normality. Differences between groups were determined with an ANOVA, and a subsequent Tukey's multiple comparison post-hoc test was performed. The level of statistical significance was set at ∗p < 0.05 and ∗∗p < 0.01.

## 3 Results

### 3.1 Metabolomic profiling of PG and SMG cells revealed significant changes after H_2_O_2_ exposure

**Fig. 1:**
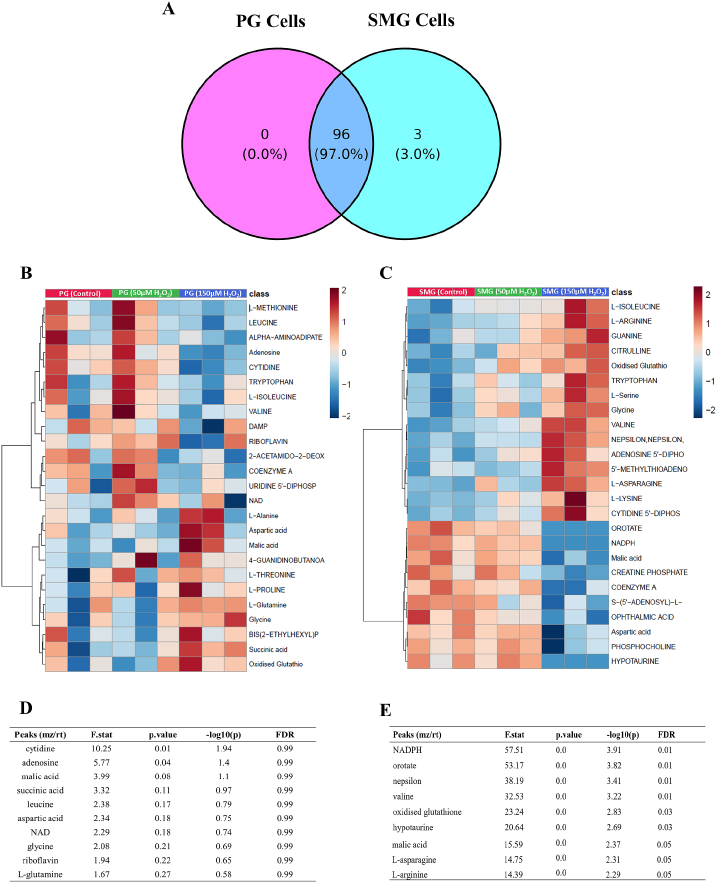
**(A)** Venn diagram of all **the** metabolites detected in PG and SMG cells. **(B, C)** Hierarchical clustering heatmaps showing the 25 most significantly affected metabolites in PG and SMG cells, respectively, detected in 50 μM and 150 μM H_2_O_2_-treated samples compared to controls. **(D, E)** The significance of the intensity (peak area) levels of the top ten metabolites identified in PG and SMG cells, respectively. Analysis with three separate seedings of different cell passages (n = 3), each with two technical replicates (n = 2).

We used LC‒MS to identify metabolites of interest in PG and SMG acinar cells treated with 50 μM or 150 μM H_2_O_2_ for 24 h, as well as in untreated control cells. All the metabolites detected in PG (n = 96) were shared with SMG (n = 99), as illustrated in the Venn diagram (Fig. 1A). Only three metabolites, including 4-acetamidobutanoate, NADPH, and orotate, were specifically detected in SMG cells in response to H_2_O_2_ treatment, while remaining below the limit of detection in PG cells. The heatmaps (Fig. 1B and C) display the metabolites whose levels were significantly affected by H_2_O_2_ exposure in PG and SMG cells, respectively. Fig. 1D highlights the top 10 metabolites in PG cells with the most significant changes in their levels, as determined by ANOVA and post-hoc analysis. Notably, cytidine showed the most significant alteration (p = 0.01), followed by metabolites such as malic acid and succinic acid, which also displayed notable changes across the control, 50 μM, and 150 μM H_2_O_2_-treated groups. Similarly, Fig. 1E presents the top 10 most significantly affected metabolites in SMG cells, where NADPH demonstrated the most significant response (p < 0.01). Other significantly affected metabolites in SMG cells included orotate, valine, and oxidized glutathione, highlighting key changes in oxidative stress-related pathways.

**Fig. 2:**
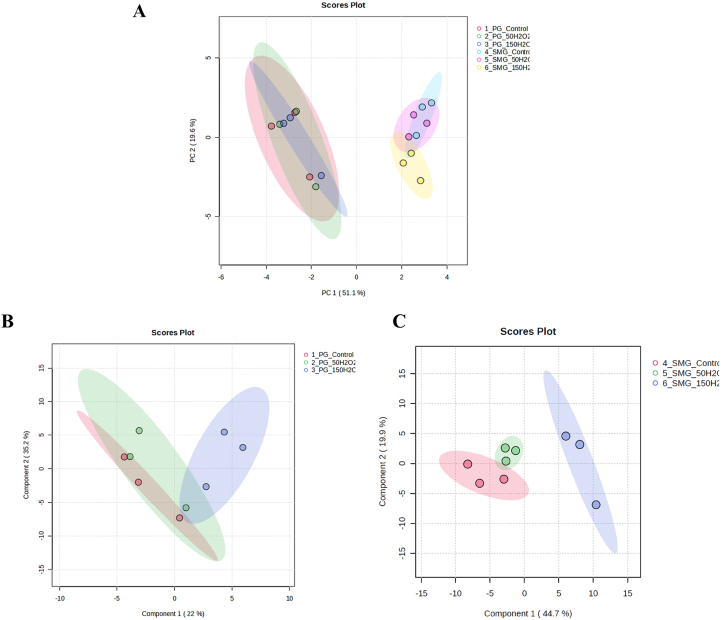
**(A)** PCA score plots for metabolic separation among the PG and SMG groups under control, 50, and 150 μM H_2_O_2_ treatments. **(B, C)** PLS-DA score plots for the metabolic separation of control, 50, and 150 μM H_2_O_2_ treatments in PG and SMG cells, respectively. Analysis with three separate seedings of different cell passages, biological replicates (n = 3), each with two technical replicates (n = 2).

We further performed two types of analyses, Principal Component Analysis (PCA) and Partial Least Squares Discriminant Analysis (PLS-DA), to compare the metabolite profiles of the two cell lines. Fig. 2A shows the PCA score plots for PG and SMG salivary gland acinar cells under control, 50 μM H_2_O_2_, and 150 μM H_2_O_2_ treatments. The two salivary gland cell lines formed clearly separate clusters, indicating distinct overall metabolic profiles between PG and SMG cells. For PG cells, metabolites from the control, 50 μM H_2_O_2_, and 150 μM H_2_O_2_ treatments converged in similar regions of the score plots, indicating minimal differences in metabolite profiles among these groups. In contrast, for SMG cells, metabolites from the control and 50 μM H_2_O_2_ treatments clustered closely, suggesting similar metabolic profiles. However, metabolites from the 150 μM H_2_O_2_ treatment group formed a distinct cluster, indicating a divergent metabolic response compared to the control and 50 μM H_2_O_2_ groups. To further explore the differences among sample clusters, we employed the PLS-DA model. The PLS-DA score plot for PG cells indicated partial overlap of the control and the two treatment groups in certain regions of the plot. The control and 50 μM H_2_O_2_ treatments converged more closely, while the 150 μM H_2_O_2_ treatment formed a distinct and separate cluster, highlighting a significant metabolic shift (Fig. 2B). Similarly, the PLS-DA score plot for SMG cells revealed that the control and 50 μM H_2_O_2_ treatments clustered closely together, indicating similar metabolic profiles. In contrast, compared with the other groups, the 150 μM H_2_O_2_ treatment group formed a well-separated cluster, emphasizing a significant metabolic shift (Fig. 2C).

Fold change analysis (Supplementary Material, Fig. S1) showed individual metabolites contributing to the observed group separations. These pairwise comparisons were performed between the control and each treatment condition within both the PG and SMG cell lines. In PG cells, limited fold changes between control and 50 μM H_2_O_2_ groups are consistent with the close clustering seen in the multivariate plots, whereas the more pronounced changes in the 150 μM H_2_O_2_ group correspond with its distinct separation. Similarly, in SMG cells, the minimal differences between control and 50 μM H_2_O_2_ groups support their overlapping clusters, whereas the clear divergence of the 150 μM H_2_O_2_ group in both PCA and PLS-DA is reflected by a larger number and magnitude of metabolite changes.

### 3.2 Functional analysis of significantly affected metabolites detected in SMG and PG acinar cell lines exposed to different concentrations of H_2_O_2_

**Fig. 3:**
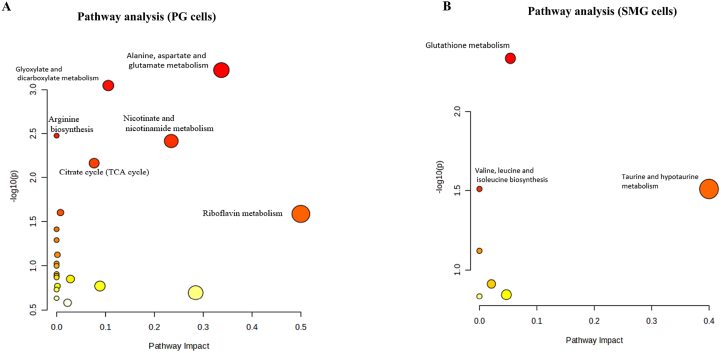
Pathway analysis of significantly affected metabolites in **(A)** PG cells and **(B)** SMG cells exposed to H_2_O_2_. The pathway impact is plotted on the x-axis, while the y-axis represents the pathway enrichment. Each node represents a distinct pathway, with larger sizes and darker colors (yellow to red) representing higher pathway impact values and higher pathway enrichment, respectively.

To understand how the affected metabolites in SMG and PG cells exposed to different concentrations of H_2_O_2_ contribute to various metabolic pathways, we performed pathway analysis according to the Kyoto Encyclopedia of Genes and Genomes (KEGG) database. In PG cells, the alanine, aspartate, glutamate metabolism pathway, glyoxylate and dicarboxylate metabolism pathway, and the TCA cycle pathway all showed high impact and significance (Fig. 3A). In SMG cells, the antioxidant defense pathway (glutathione metabolism) was the most significantly impacted pathway (Fig. 3B). This highlights distinct metabolic pathway alterations in the two cell lines, possibly reflecting differences in their metabolic responses to oxidative stress.

**Fig. 4:**
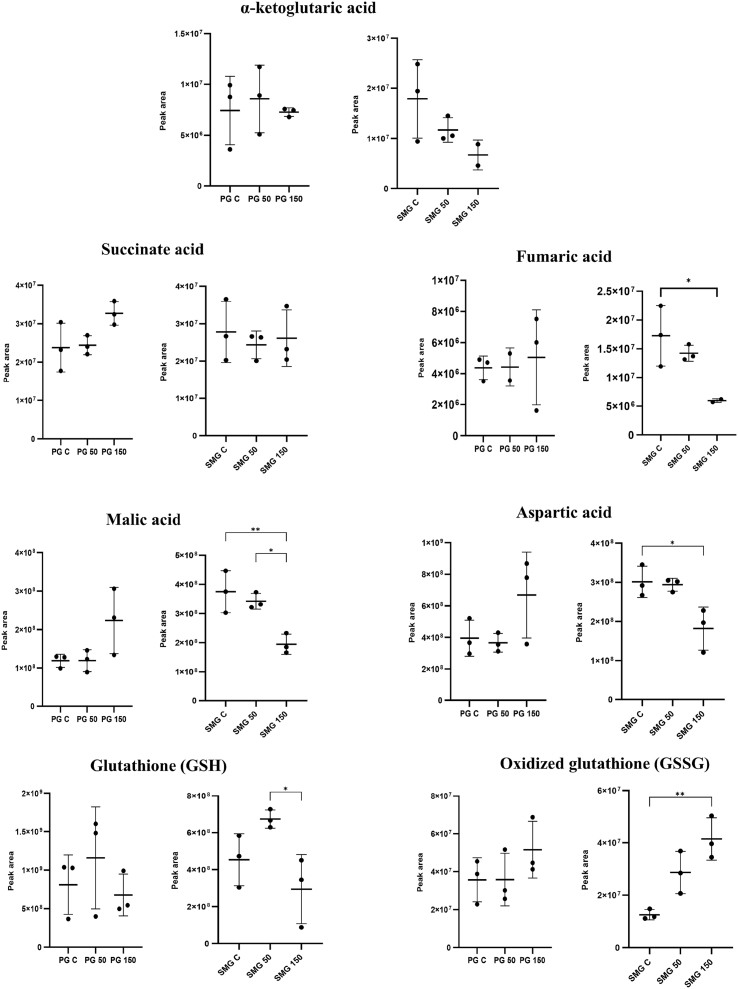
Detected metabolites showing different peak areas across H_2_O_2_ exposure conditions in PG and SMG acinar cells. Dot plot with error bars representing the mean ± standard deviation. Statistical analysis was performed using ANOVA followed by Tukey's multiple comparison post hoc test, ∗p < 0.05; ∗ ∗p < 0.01. Analysis with three separate seedings of different cell passages, biological replicates (n = 3), each with two technical replicates (n = 2).

We identified several metabolites that showed significant differences in their detected levels, under different H_2_O_2_ exposure in PG and SMG acinar cells (Fig. 4). These metabolites may contribute to glutamate metabolism, glutathione metabolism, and the TCA cycle. Succinic acid, fumaric acid, malic acid, and aspartic acid are critical intermediates of the TCA cycle. Three of them (fumaric, malic, and aspartic acid) significantly decreased in SMG cells under H_2_O_2_ exposure compared to controls. In contrast, PG cells displayed an increasing trend in these metabolites. However, due to high biological variation observed between replicates in the PG cell line, these elevations did not reach statistical significance. α-Ketoglutaric acid, a precursor of succinic acid in the TCA cycle, exhibited a decreasing in both PG and SMG acinar cells under 150 μM H_2_O_2_ treatment compared to the control, although the change was not statistically significant. Glutathione (GSH) and oxidized glutathione (GSSG), key metabolites in glutathione metabolism, exhibited significant concentration-dependent changes in response to H_2_O_2_ exposure in SMG cells. In SMG cells, GSH levels were not significantly increased at 50 μM but were significantly decreased at 150 μM compared to controls. In contrast, while PG cells exhibited similar patterns, no statistically significant changes were observed at either concentration. Conversely, GSSG displayed an increasing at 150 μM, which was also significant for SMG cells. The decline in GSH levels after exposure to the highest concentration, along with the corresponding increase in GSSG levels, particularly in SMG cells, indicates heightened oxidative stress and an active glutathione redox cycle.

### 3.3 Validation of succinate levels in PG and SMG cells under varying H_2_O_2_ concentrations

**Fig. 5:**
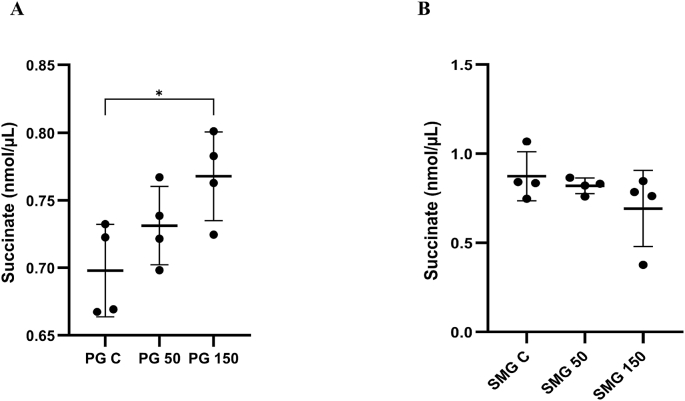
**(A)** Concentrations of succinate under control, 50, and 150 μM H_2_O_2_ treatments in PG cells and **(B)** SMG cells. Dot plots with error bars represent the mean ± standard deviation. Statistical analysis was performed using ANOVA followed by Tukey's multiple comparison post hoc test, ∗p < 0.05. Analysis with four separate seedings of different cell passages, biological replicates (n = 4).

To explore how oxidative stress alters mitochondrial metabolism in different salivary gland cells and to validate our metabolomics results, we measured succinate levels in PG and SMG cells after exposure to 50 and 150 μM H_2_O_2_ compared to untreated controls. In PG cells, succinate levels increased significantly in a dose-dependent manner, suggesting enhanced mitochondrial activity or a disruption in the TCA cycle under oxidative conditions (Fig. 5A). In contrast, SMG cells exhibited a not significant decrease in succinate levels with increasing H_2_O_2_ concentrations (Fig. 5B). This distinct metabolic response may reflect impaired mitochondrial function or differences in oxidative stress tolerance between the two gland types.

## 4 Discussion

In this study, we used H_2_O_2_ exposure to induce oxidative stress, as it is one of the most common methods for generating oxidative stress in various cellular models [35]. We subsequently examined its effect on metabolite expression in salivary gland acinar cell lines and compared the metabolite profiles between SMG and PG cells.

We identified three metabolites, 4-acetamidobutanoate, NADPH, and orotate that were exclusively detected in SMG cells following H_2_O_2_ treatment. NADPH is a key cofactor in antioxidant defense and is essential for maintaining redox homeostasis through the regeneration of reduced glutathione [36]. 4-Acetamidobutanoate is a metabolic intermediate in the biosynthesis of γ-aminobutyric acid (GABA), which has been shown to increase cell viability, decrease ROS production, and upregulate the expression of antioxidant genes in renal cortex cell culture [37]. Orotate is a key intermediate in pyrimidine metabolism and has been implicated in antioxidant defense [38]. In contrast to PG cells, the specific presence of these unique metabolites (Orotate, NADPH, and 4-acetamidobutanoate) in SMG cells suggests a more robust or distinct antioxidant response of SMG cells to H_2_O_2_-induced oxidative stress.

One of the metabolites identified in our metabolomics analysis was succinate. The opposite expression pattern of succinate in PG and SMG cells was showed, with an increase in succinate levels in PG cells. This finding was validated by use of a succinate detection kit. Succinate was validated because it links mitochondrial metabolism, redox balance, and oxidative stress. Altered succinate levels can indicate shifts in cellular metabolic activity and stress responses, underscoring its relevance as a biologically significant target for validation. Here, similar to the metabolomics data, a significant increase in succinate levels in PG cells after H_2_O_2_ exposure compared to the control was detected. In contrast, there was no significant reduction in succinate levels in SMG cells. Moreover, we observed non-significantly reduced levels of α-ketoglutaric acid, a precursor of succinic acid in the TCA cycle, in both PG and SMG acinar cells exposed to 150 μM H_2_O_2_ compared to the control.

Succinate is a key contributing factor to reverse electron transport, a process in which electrons flow backward through complex I of the mitochondrial electron transport chain, which can enhance ROS production at complex I [39]. It has been shown that nearly half of the ROS generated by brain mitochondria under certain conditions are driven by succinate-dependent RET [40]. Serum and plasma succinate levels have been shown to be elevated in patients with lung cancer and head and neck cancer, respectively, compared to healthy individuals, suggesting their potential as prognostic biomarkers [41,42]. Furthermore, a study on the salivary metabolite profile in Sjögren's disease patients revealed increased levels of succinate compared to the control group [43]. Both Sjögren's disease and head and neck cancer involve salivary gland dysfunction and elevated oxidative stress, which may contribute to altered succinate metabolism. The elevated succinate levels observed in PG cells but not in SMG cells may contribute to mitochondrial ROS production via RET in PG cells. Interestingly, our findings indicate that distinct patterns of succinate accumulation are linked to oxidative stress in PG and SMG acinar cells following H_2_O_2_ exposure.

Furthermore, we observed that fumaric acid, malic acid, and aspartic acid, key intermediates of the TCA cycle, were not significantly increased in PG cells but were significantly decreased in SMG cells following H_2_O_2_ exposure compared to the control. The fumarate–malate–oxaloacetate pathway, initiated by succinate, is considered one of the essential metabolic pathways for maintaining mitochondrial function and the cellular redox balance [21]. Therefore, the observed reduction in fumaric and malic acid levels in SMG cells compared to PG cells under H_2_O_2_-induced oxidative stress may be attributed to lower succinate levels in SMG cells under H_2_O_2_ exposure. Moreover, the observed reduction in fumaric and malic acid levels in SMG cells compared to PG cells under H_2_O_2_-induced oxidative stress may be attributed to lower succinate levels in SMG cells under H_2_O_2_ exposure. This coordinated suppression of TCA intermediates suggests a protective metabolic dampening or redirection in SMG cells. Unlike PG cells, which accumulate succinate and trigger ROS-generating RET [39], SMG cells appear to restrict mitochondrial flux to limit oxidative damage. Furthermore, the detection of 4-acetamidobutanoate in SMG cells implies a potential redirection of carbon flux towards the GABA pathway (GABA shunt). The GABA shunt is an alternative metabolic route that converts glutamate to succinate via GABA, effectively bypassing the ROS-sensitive α-ketoglutarate dehydrogenase complex [44]. This metabolic rerouting allows the cells to bypass the ROS-sensitive steps of the TCA cycle while maintaining energy homeostasis and supporting antioxidant defense [45]. It has been demonstrated that excess fumarate accumulates in renal cancer cells, and it has been suggested that fumarate can bind directly to the antioxidant GSH, forming a modified compound known as succinated glutathione (GSF) [46]. GSF can act as a substrate for glutathione reductase, leading to NADPH consumption and increased mitochondrial ROS production. Interestingly, our results showed high levels of NADPH in SMG cells, whereas NADPH remained below the limit of detection in PG cells. Furthermore, while fumaric acid accumulated in PG cells, it was significantly reduced in SMG cells. This divergence specifically the clearance of TCA metabolites and the preservation of NADPH suggests that SMG cells mount a stronger compensatory metabolic response to oxidative stress. In contrast, NADPH levels in PG cells remained below the limit of detection. This, combined with the retention of fumaric acid in PG cells, in contrast to the significant reduction seen in SMG cells, suggests that PG cells suffer from both antioxidant depletion and mitochondrial metabolic stagnation, rendering them highly susceptible to oxidative damage.

Aspartic acid, another TCA-linked metabolite, showed a non-significant elevation in PG cells, which is consistent with its reported accumulation under conditions of mitochondrial dysfunction [47], while it was significantly downregulated in SMG cells in response to H_2_O_2_ treatment. Upon ETC inhibition, glutamic-oxaloacetic transaminase 1 (GOT1), an enzyme that normally consumes aspartic acid to transfer electrons into the mitochondria, can reverse its activity and generate aspartic acid in the cytosol. This reversal may compensate for the loss of mitochondrial aspartate and reflects an impaired malate-aspartate shuttle. One may speculate that the trend toward elevated aspartic acid levels in PG cells may indicate a breakdown in the mitochondrial redox balance, where GOT1 reverses its activity to produce aspartate in the cytosol as temporary compensation for impaired electron transfer.

In mammalian cells, cycling between GSH and oxidized glutathione (GSSG) is essential for detoxifying ROS to protect cells from oxidative damage [48]. Our KEGG pathway analysis showed enrichment of glutamate and GSH metabolism in PG and SMG cells exposed to H_2_O_2_, respectively. Additionally, we observed a trend toward reduced GSH levels in both cell lines when exposed to a high concentration of H_2_O_2_ compared to the control. Moreover, the level of GSSG, which is known to be potentially cytotoxic and accumulates as a result of free radical neutralization, was increased in both cell lines under H_2_O_2_ exposure; however, the elevation was statistically significant only in SMG cells. The accumulation of GSSG under oxidative stress conditions highlights the imbalance between ROS production and antioxidant defense mechanisms in both cell lines. It has been reported that excessive oxidative stress leads to GSSG accumulation and inhibits glutathione reductase activity, especially under conditions where NADPH is limited [49].

NADPH is a vital cofactor for glutathione reductase, which recycles GSSG back to GSH [50]. It has been shown that NADPH not only supports GSH recycling but also plays a central role in mitochondrial function and antioxidant capacity via the pentose phosphate pathway (PPP) and malic enzyme activity [51]. Of note, our results showed a disparity in NADPH availability, suggesting a key difference in how SMG and PG cells manage redox homeostasis. The higher NADPH levels in SMG cells likely support more efficient GSSG reduction, thereby maintaining redox balance and limiting ROS-mediated damage. Conversely, in PG cells, the absence of detectable NADPH may hinder GSSG recycling, resulting in prolonged oxidative stress and potential cytotoxic effects.

The PCA and PLS-DA analysis resulted in clearly separated clusters for the two salivary gland cell lines induced by H_2_O_2_, indicating distinct metabolic responses. In PG cells, the metabolic profiles remained relatively stable across H_2_O_2_ exposure. In contrast, SMG cells showed a marked shift at 150 μM H_2_O_2_, suggesting a stronger metabolic response to oxidative stress. In a previous study, we demonstrated that H_2_O_2_ exposure differentially affects the protein profiles of PG and SMG acinar cells, with SMG cells exhibiting higher basal levels of antioxidant proteins and proteins involved in metabolic pathways and greater viability compared to PG cells [29]. Along with previous findings, our results suggest that SMG cells have a different and possibly more flexible metabolism to handle oxidative stress compared to PG cells. The distinct clustering patterns in the PCA and PLS-DA analyses, along with a clear metabolic shift in SMG cells at 150 μM H_2_O_2_, higher NADPH levels, and less succinate accumulation compared to PG cells, all suggest that SMG cells adapt better to stress.

The differential vulnerability of salivary glands is largely attributed to their cellular composition. The PG is comprised predominantly of serous acinar cells, which are highly radiosensitive, whereas the SMG contains a significant proportion of mucous cells, which are relatively radioresistant [52]. This fragility of acinar cells aligns with the metabolic inability to manage the stress we identified in PG cells. Similarly, in chronic conditions like Sjögren's disease [53] and diabetes [54], where oxidative stress is a key driver of glandular dysfunction, the specific metabolic imbalances we observed, such as succinate accumulation and NADPH depletion, may play a pivotal pathogenic role. These insights suggest that new therapies designed to support mitochondrial function could help prevent permanent gland failure in these patients.

**Fig. 6:**
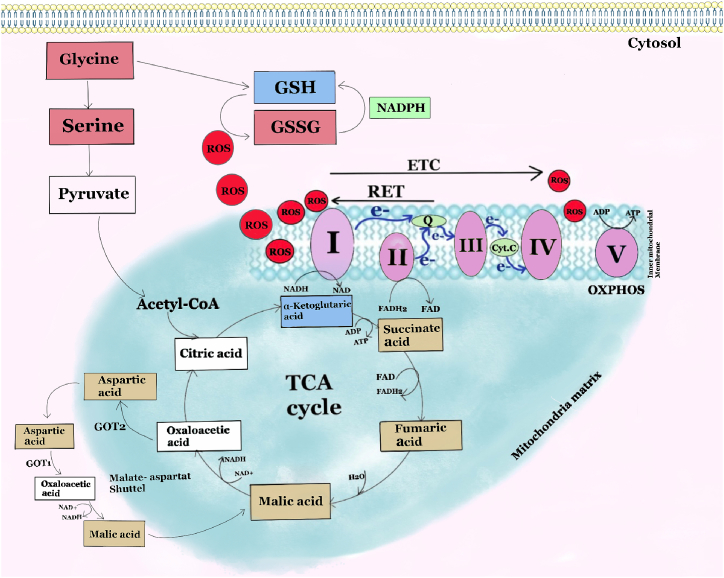
The image summarizes the metabolic interplay between glutathione (GSH) metabolism and the TCA cycle in the context of oxidative stress in salivary parotid gland (PG) and submandibular gland (SMG) acinar cells. Through a series of enzymatic steps, the tricarboxylic acid (TCA) cycle generates the reducing agents NADH and FADH_2_, which supply electrons to the mitochondrial electron transport chain (ETC). As these electrons move through ETC complexes, they create the mitochondrial membrane potential necessary for ATP synthesis, a process known as oxidative phosphorylation (OXPHOS). Within the mitochondria, reactive oxygen species (ROS) are predominantly generated at complexes I and III of the ETC, particularly during reverse electron transport (RET). In RET, a portion of electrons from reduced ubiquinol are pushed backward toward complex I by the mitochondrial membrane potential. Excessive ROS can impair mitochondrial function and alter the abundance of TCA intermediates such as succinate, malate, and fumarate, which tend to accumulate under oxidative stress, as observed in PG acinar cells. In response to elevated ROS levels, glycine, an essential precursor for GSH synthesis, supports the production of GSH, a major antioxidant that neutralizes ROS. During the neutralization process, GSH is converted to its oxidized form (GSSG) and subsequently regenerated using NADPH. Additionally, the TCA cycle is linked to the malate-aspartate shuttle and transamination reactions mediated by glutamic-oxaloacetic transaminases 1 and 2 (GOT1 and GOT2), which contribute to maintaining the cellular redox balance and metabolic homeostasis. The metabolites highlighted in red boxes are increased, and the metabolites highlighted in blue boxes are decreased in both PG and SMG acinar cells following H_2_O_2_ exposure. Metabolites in brown boxes are elevated in PG cells but decreased in SMG cells after H_2_O_2_ treatment. The metabolite in the green box is detected only in SMG cells and shows increased levels in response to elevated H_2_O_2_. Metabolites in white boxes were not detected in our data.

Overall, these results highlight potential metabolic biomarkers for detecting salivary gland dysfunction and reveal key differences in how the two salivary gland cell types respond to stress. Nonetheless, further studies are needed to elucidate the metabolic pathways underlying the distinct redox and stress responses in SMG and PG cells (Fig. 6).

## 5 Conclusion

In conclusion, our study identified three metabolites, 4-acetamidobutanoate, NADPH, and orotate, that were uniquely detected in SMG cells following H_2_O_2_ exposure, highlighting their potential roles in antioxidant defense. Moreover, PG and SMG salivary gland cells responded differently to H_2_O_2_-induced oxidative stress. SMG cells exhibited higher NADPH levels, less succinate accumulation, and greater metabolic adaptability in favor of enhanced antioxidant defense and redox homeostasis. In contrast, PG cells showed increased levels of TCA cycle metabolites such as succinic acid, malic acid, and fumaric acid. Based on these findings, we propose that intracellular succinate levels, NADPH availability, and the GSH/GSSG ratio could serve as a valuable panel of potential biomarkers. Monitoring these metabolic indicators may provide critical insights into the differential susceptibility of salivary glands during irradiation, aiding in the development of targeted gland-preservation strategies.

These findings reveal differences in metabolic responses to oxidative stress and point to the need for further studies to identify the specific pathways involved in redox regulation and stress adaptation in salivary gland acinar cells. This is important for understanding salivary gland-specific vulnerabilities and for the future development of targeted therapies for oxidative stress-related dysfunction. However, as this study utilized immortalized rat cell lines to map fundamental metabolic shifts, future translational studies are warranted. Validating these metabolic signatures in human salivary gland tissue biopsies or non-invasive clinical saliva samples will be essential to confirm their utility as diagnostic tools and to guide the development of gland-sparing therapies.

## Funding

The research received its main funding from internal resources, with supplementary support provided by UNIFOR-FriMed and Stiftelsen for tannlegevitenskapens fremme.

## CRediT authorship contribution statement

**Golnaz Golnarnik:** Conceptualization, Formal analysis, Investigation, Methodology, Validation, Visualization, Writing – original draft, Writing – review & editing. **Anni I. Nieminen:** Data curation, Formal analysis, Methodology, Visualization, Writing – review & editing. **Tine M. Søland:** Resources, Supervision, Writing – review & editing. **Hilde K. Galtung:** Conceptualization, Methodology, Resources, Supervision, Writing – review & editing. **Trude M. Haug:** Conceptualization, Funding acquisition, Project administration, Resources, Supervision, Writing – review & editing.

## Declaration of competing interest

The authors declare that they have no known competing financial interests or personal relationships that could have appeared to influence the work reported in this paper.

## Data Availability

Data will be made available on request.
