# Supplementary material for: Differential metabolic responses to hydrogen peroxide-induced oxidative stress in parotid and submandibular gland acinar cell lines
Source: Biochem Biophys Rep. 2026 Apr 17;46:102596. doi: 10.1016/j.bbrep.2026.102596 (PMC13101717; doi:10.1016/j.bbrep.2026.102596)
Supplement: Multimedia component 1 [file mmc1.docx]

**Supplementary material**


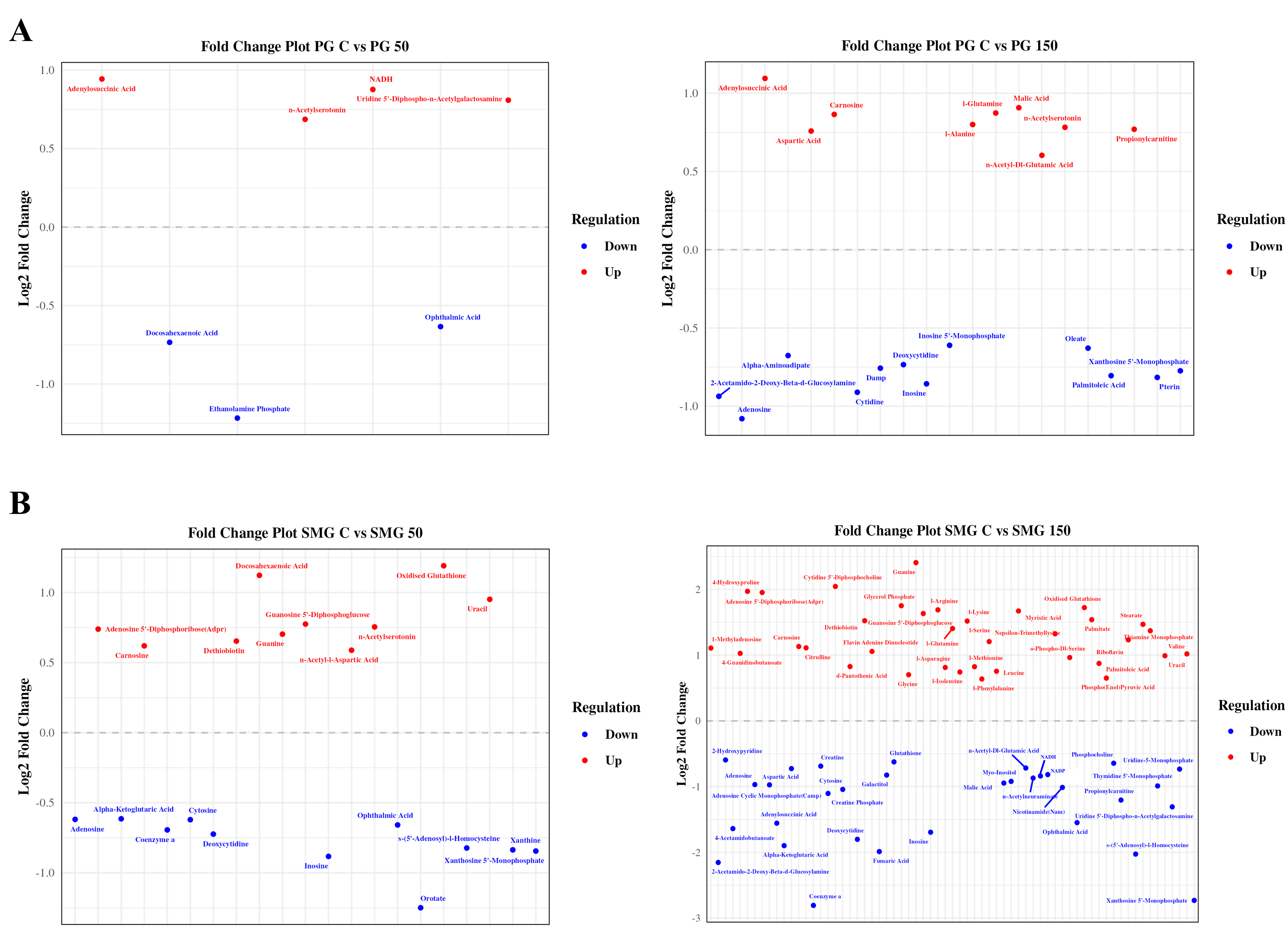
**Figure S1:** **(A)** Fold change plots comparing the untreated control (C) with 50 µM (left) and 150 µM (right) H₂O₂-treated samples in PG cells. **(B)** Fold change plots comparing the untreated control (C) with 50 µM (left) and 150 µM (right) H₂O₂-treated samples in SMG cells. Red dots indicate significantly upregulated metabolites, while blue dots indicate significantly downregulated metabolites. Fold change threshold ≥ 1.5.

**Table S1:** Chromatographic Gradient Protocol Separation was performed on a SeQuant ZIC-pHILIC column (2.1x100 mm, 5-μm) at a flow rate of 0.100 mL/min. Mobile Phase A = 20 mM ammonium hydrogen carbonate (pH 9.4); Mobile Phase B = Acetonitrile.

| **Time (min)** | **Mobile Phase A (%)** | **Mobile Phase B (%)** | **Phase Description** |
| --- | --- | --- | --- |
| **0.0 – 2.0** | 20 | 80 | Isocratic hold |
| **2.0 – 17.0** | 20 to 80 | 80 to 20 | Linear gradient |
| **17.0 – 24.0** | 20 | 80 | Re-equilibration |

**Table S2:** Mass Spectrometry Source Settings Data were acquired on a Q-Exactive Orbitrap using a Heated Electrospray Ionization (H-ESI) source.

| **Parameter** | **Setting** |
| --- | --- |
| Scan Range | 55 – 825 m/z |
| Resolution (MS1) | 35,000 |
| Spray Voltage (Positive Mode) | 4250 V |
| Spray Voltage (Negative Mode) | 3250 V |
| Sheath Gas Flow | 25 AU |
| Auxiliary Gas Flow | 15 AU |
| Sweep Gas Flow | 0 AU |
| Capillary Temperature | 275°C |
| S-lens RF Level | 50.0 |
